# Mechanisms of salt tolerance in habanero pepper plants (*Capsicum chinense* Jacq.): Proline accumulation, ions dynamics and sodium root-shoot partition and compartmentation

**DOI:** 10.3389/fpls.2014.00605

**Published:** 2014-11-12

**Authors:** Emanuel Bojórquez-Quintal, Ana Velarde-Buendía, Ángela Ku-González, Mildred Carillo-Pech, Daniela Ortega-Camacho, Ileana Echevarría-Machado, Igor Pottosin, Manuel Martínez-Estévez

**Affiliations:** ^1^Unidad de Bioquímica y Biología Molecular de Plantas, Centro de Investigación Científica de YucatánYucatán, México; ^2^Centro Universitario de Investigaciones Biomédicas, Universidad de ColimaColima, México; ^3^Unidad de Ciencias del Agua, Centro de Investigación Científica de YucatánYucatán, México

**Keywords:** salt tolerance, pepper, roots, proline accumulation, sodium compartmentalization, potassium retention, ion fluxes, H^+^-ATPase

## Abstract

Despite its economic relevance, little is known about salt tolerance mechanisms in pepper plants. To address this question, we compared differences in responses to NaCl in two *Capsicum chinense* varieties: Rex (tolerant) and Chichen-Itza (sensitive). Under salt stress (150 mM NaCl over 7 days) roots of Rex variety accumulated 50 times more compatible solutes such as proline compared to Chichen-Itza. Mineral analysis indicated that Na^+^ is restricted to roots by preventing its transport to leaves. Fluorescence analysis suggested an efficient Na^+^ compartmentalization in vacuole-like structures and in small intracellular compartments in roots of Rex variety. At the same time, Na^+^ in Chichen-Itza plants was compartmentalized in the apoplast, suggesting substantial Na^+^ extrusion. Rex variety was found to retain more K^+^ in its roots under salt stress according to a mineral analysis and microelectrode ion flux estimation (MIFE). Vanadate-sensitive H^+^ efflux was higher in Chichen-Itza variety plants, suggesting a higher activity of the plasma membrane H^+^-ATPase, which fuels the extrusion of Na^+^, and, possibly, also the re-uptake of K^+^. Our results suggest a combination of stress tolerance mechanisms, in order to alleviate the salt-induced injury. Furthermore, Na^+^ extrusion to apoplast does not appear to be an efficient strategy for salt tolerance in pepper plants.

## Introduction

The excess of soluble salts in soil, particularly NaCl, causes three types of stresses in plants: osmotic, ionic, and oxidative. These stresses reduce absorption and induce a massive efflux of water and ions (K^+^) in plant cells, resulting in water, and nutritional imbalances. The accumulation of Na^+^ to toxic concentrations and the production of reactive oxygen species (ROS) reduce the growth, yield, and production of economically important crops, such as cereals and vegetables (Munns and Tester, 2008; Bojórquez-Quintal et al., 2012). Plants in relation to salt can be classified into two groups: halophytes (growth stimulated at moderate and tolerant to high salinity) and glycophytes, which display a suppressed growth in a saline environment (Flowers and Colmer, 2008; Ruan et al., 2010). In halophytes, various adaptive mechanisms to tolerate high levels of salt have been identified and intensively studied (Ruan et al., 2010; Adolf et al., 2013; Shabala, 2013). Unfortunately, some of these mechanisms may not be directly transferred to crop plants, which are mostly glycophytes (Zhang and Shi, 2013). Yet, several crops are relatively salt resistant, and there are also substantial differences in the salt tolerance between nearly isogenic varieties within the same plant species. Salt tolerance is a complex trait controlled by many genes and involves various biochemical and physiological mechanisms. The fine tuning of these mechanisms is necessary to achieve a significant increase in tolerance to salt (Zhang and Shi, 2013; Adem et al., 2014).

Proline is the most common compatible osmolyte in plants and has therefore been extensively studied. The accumulation of this amino acid is an important regulatory mechanism under osmotic stress (Huang et al., 2013). Proline is a multifunctional amino acid (Szabados and Savouré, 2009). In many plant species, the accumulation of proline has been associated with tolerance to salt stress and has even been used as a marker to select tolerant genotypes (Ashraf and Harris, 2004). However, a negative correlation between the accumulation of proline and salt tolerance has also been reported, indicating discrepancies in its function (Lutts et al., 1999; Chen et al., 2007a). Proline accumulation is made possible by the increase in the expression and activity of the synthesis enzymes (Δ-pyrroline-5-carboxylate synthetase, P5CS; Δ-pyrroline-5-carboxylate reductase, P5CR) or by the decrease in the degradation enzymes, proline dehydrogenase or proline oxidase (PDH or POX), and P5C dehydrogenase (P5CDH) (Huang et al., 2013). Under salt stress, the *P5CS1* and *PDH* genes are positively and negatively regulated, respectively (Kishor et al., 2005; Verslues and Sharma, 2010; Jaarsma et al., 2013). Similarly, the overexpression of the *P5CS* gene increases proline synthesis under salt stress and improves tolerance to salt (Kishore et al., 1995; Hmida-Sayari et al., 2005).

The roots are the first site of contact with high concentrations of Na^+^ in the soil and therefore of the uptake or absorption of salt. Na^+^ influx is mediated by non-selective cation channels (NSCC), high-affinity K^+^ transporters (HKTs), and low-affinity cation transporters (LCT) in the root epidermal cells (Apse and Blumwald, 2007; Plett and Moller, 2010; Maathuis, 2014). Na^+^ is then transported radially toward the root xylem via the apoplast and symplast. After being loaded into the xylem, Na^+^ is finally transported to the shoots by xylem flow (Adams and Shin, 2014). In contrast to halophytes, Na^+^ is not an essential element for most plants and becomes highly toxic at high concentrations, particularly in the aerial parts of the plant. Therefore, it is necessary to maintain efficient control of Na^+^ content and intracellular compartmentalization in plant tissues. The high-affinity potassium transporters (HKTs), the Na^+^/H^+^ SOS1 (salt overly sensitive) antiporters on the plasma membrane and the intracellular NHX antiporters (Na^+^/H^+^) are transporters involved in the Na^+^ homeostasis (Almeida et al., 2013; Adams and Shin, 2014).

The regulation of K^+^ homeostasis is essential for plant adaptation to biotic and abiotic stresses. This adaptation is associated with the wide range of functions in which K^+^ participates (Anschütz et al., 2014; Demidchik, 2014; Shabala and Pottosin, 2014). Recently, K^+^ retention in the cells of roots and leaves has been identified as an important trait for salt tolerance. A strong negative correlation between the magnitude of salt-induced K^+^ loss and salt tolerance, observed in various crop species, suggested K^+^ retention as a selection criterion between salt tolerant and sensitive varieties (Chen et al., 2005, 2007b,c; Smethurst et al., 2008; Lu et al., 2013; Wu et al., 2013; Bonales-Alatorre et al., 2013a). Furthermore, it has been observed that the exogenous administration of organic compounds and divalent cations prevents K^+^ efflux (Cuin and Shabala, 2005, 2007a,b; Shabala et al., 2006; Zhao et al., 2007; Chen et al., 2007a; Zepeda-Jazo et al., 2008). Efficient control of membrane potential due to the H^+^-ATPase activity was shown to be important for the salt tolerance in several species (Chen et al., 2007b; Cuin et al., 2008; Hariadi et al., 2011; Bose et al., 2013, 2014). A more negative membrane potential during salt stress reduces the driving force for the K^+^ loss and facilitates the K^+^ absorption, thus allowing plants to retain K^+^ in the cytosol (Chen et al., 2007b; Bose et al., 2013). Likewise, the H^+^-ATPase activity is essential to fuel Na^+^/H^+^ exchangers in the plasma membrane (SOS1). At the same time, a higher activity of the H^+^ pump consumes a large amount of ATP, hence has a higher energetic cost (Malagoli et al., 2008). Thus, keeping electrochemical gradients for physiologically important cations across the plasma membrane could present an energetic burden, so this tolerance mechanism cannot be considered permanent and may be used as a temporary solution at early times after the onset of the salt stress (Bose et al., 2013, 2014).

Peppers (*Capsicum* spp.) are an economically important genus of the *Solanaceae* family, which also includes tomatoes and potatoes. Among the 32 species native to America, *C. annuum L., C. baccatum L., C. frutescens L., C. pubescens L.*, and *C. chinense* Jacq. are cultivated (Moscone et al., 2007; Perry et al., 2007). Overall, pepper plants are grown around the world because of their adaptation to different agro-climatic regions and their wide variety of shapes, sizes, colors, and pungencies of the fruit (Qin et al., 2014). However, these plants are sensitive to various biotic stresses, such as viruses and Oomycetes and abiotic factors such as drought and salinity. In fact, pepper plants are considered moderately sensitive, sensitive or highly susceptible to salt stress (Maas and Hoffman, 1977; Aktas et al., 2006). Nevertheless, despite their economic importance as a horticultural species, very little is known about the mechanisms of tolerance to high salt concentrations. To contribute to the understanding of salt stress in species of economic importance such as peppers, the difference in salt sensitivity of two varieties of the species *C. chinense* Jacq, commonly known as habanero pepper, was evaluated in this study. Furthermore, possible mechanisms of salt stress tolerance for the two varieties were addressed by electrophysiological studies using selective microelectrodes (MIFE) and by subcellular localization of Na^+^ using fluorescent indicators.

## Materials and methods

### Plant material and growth conditions

Habanero pepper (*C. chinense* Jacq.) seeds of the Chichen-Itza (Seminis^®^) and Rex (Mayan Chan obtained in CICY) varieties were used in this study. To disinfect the seeds, they were rinsed in 80% ethanol (v/v) for 5 min and washed continuously with sterile water. Seeds were then incubated with 30% (v/v) sodium hypochlorite (Cloralex 5% NaOCl) and Tween (1 drop) for 15 min. Washes were continuous, and the seeds were kept in sterile water for 48 h at 4°C in the dark. After stratification, seeds of both varieties were incubated (in the dark) in Petri dishes with filter paper moistened with sterile water until the emergence of the radicle.

For the hydroponic experiments, germinated seeds were transferred to plastic containers with vermiculite moistened with a Hoagland solution to a fifth of its ionic strength (H1/5). Seeds were incubated under photoperiods of 16/8 h light/dark at 25°C. The light intensity was 123 μ mol m^−2^ s^−1^. Seedlings were irrigated for 45 days with a sterile water solution and H1/5 with 7-day intervals. The modified Hoagland solution at one-fifth of its ionic strength (H1/5) contained the following: 1.2 mM KNO_3_, 0.8 mM Ca(NO_3_)_2_, 0.2 mM KH_2_PO_4_, 0.2 mM MgSO_4_, 50 μM CaCl_2_, 12.5 μM H_3_BO_3_, 1 μM MnSO_4_, 1 μM ZnSO_4_, 0.5 μM CuSO_4_, 0.1 μM (NH_4_)_6_Mo_7_O_24_, 0.1 μM NaCl, and 10 μM Fe-EDTA, pH 6.8.

For electrophysiological experiments and subcellular Na^+^ localization, seeds with radicles were transferred to Petri dishes containing modified Gamborg-B5 growth medium (Sales B5, Sigma) at half ionic strength (B5/2). B5/2 medium was supplemented with 0.5% sucrose (w/v) and 1% agar (w/v). The pH was adjusted to 5.8. Seedlings 10 days of age with a primary root 8–10 cm in length were used for this experiment.

### NaCl stress treatment

Forty–five- to fifty-day-old seedlings (growing in vermiculite) of Rex and Chichen-Itza varieties were used. After pre-treatment with a solution of H1/5 for 7 days to avoid mechanical damage, seedlings homogeneous in size were selected. Three replicates of 10 seedlings of each variety were subjected to 7 days of treatment at concentrations of 0, 50, 100, and 150 mM NaCl in H1/5 solution. Treatments were performed in a culture room with photoperiods of 16/8 h light/dark at 25°C. At the end of the experiment, the seedlings were harvested and washed with sterile water to remove excess NaCl, and the fresh weight (FW), dry weight (DW), and the water content was determined by the formula (FW-DW)/FW. Each type of sample was dried in an electric oven at 60°C for 72 h. The leaves and roots were used for the determination of proline, Na^+^, and K^+^ content.

### Proline content

A modification of the method described by Bates et al. (1973) was used to determine proline content. Briefly, dry leaf and root tissues were macerated and homogenized in 10 mL of boiling water. For each reaction, 2 mL of the supernatant was mixed with 2 mL of acetic acid and 2 mL of ninhydrin. The reaction mixture was heated in a water bath at 100°C for 60 min, and the reaction was stopped in an ice bath. For extraction, 4 mL of toluene was added, and samples were mixed vigorously for 15–20 s. Samples were then set aside to allow separation of the organic and aqueous phases. The organic phase containing the chromophore was collected in a new tube, and absorbance was read at 520 nm using toluene as a blank. Proline concentration was determined from a standard curve, and concentrations were calculated based on DW.

### Sodium and potassium quantification

Samples of dried leaves and roots were weighed, and HNO_3_:H_2_O_2_ was added at a 5:1 (v:v) ratio. Microwave digestion was performed at 1200 W using a ramp of 15 min to 200°C, 10 min to 200°C, and 5 min to 170°C. Subsequently, samples were adjusted to a volume of 25 mL with water (Milli Q), and Na^+^ and K^+^ content was quantified by inductively coupled plasma atomic emission spectroscopy (ICP-AES, Thermo IRIS Intrepid II XDL, New York, USA). Standard curves were used for each element.

### MIFE technique

The net flux of K^+^ and H^+^ on the surface of the roots of the two varieties of *C. chinense* was measured non-invasively by the microelectrode ion flux estimation (MIFE) technique (Newman, 2001). For MIFE studies, seedlings grown *in vitro* with roots of 8–10 cm in length were transferred and fixed horizontally to a measuring chamber. Subsequently, 25 mL of measurement solutions were added for K^+^ (0.5 mM KCl, 0.1 mM CaCl2, 5 mM MES, 2 mM Tris base, pH 6.0) and for H^+^ (0.5 mM KCl and 0.1 mM CaCl_2_, without pH-buffer) and incubated for 1 h to allow stabilization. Two electrodes selective for K^+^ and H^+^ were used in each experiment. For salt stress treatment, NaCl solution was added to the measuring chamber to a final concentration of 150 mM. Before the experiment, the microelectrodes were filled with 0.5 mM KCl for K^+^ or 0.15 mM NaCl and 0.04 mM KH_2_PO_4_ for H^+^, and the tip of each electrode was filled with the ion-selective resin (ion-liquid exchanger, LIX Fluka, Sigma-Aldrich) for the ion measured. Two electrodes were then mounted in a micromanipulator, and located perpendicular to the root axis 20–40 μm from the mature root zone, 1–2 cm from the root apex. Measurements were initiated by moving the electrodes 50 μm back and then forth and back in a cyclic manner every 8 s. The software CHART recorded potential differences between the two measurement positions and converted them into electrochemical potential differences using the Nernst slope. Net ion fluxes were calculated using the MIFEFLUX software for cylindrical diffusion geometry.

### Localization and subcellular accumulation of sodium

Sodium Green™ indicator (S-6901, Molecular Probes, Life Technologies) was used to evaluate the subcellular localization and accumulation of sodium in the roots of *C. chinense.* Seedlings grown *in vitro* were incubated in a measurement solution (0.5 mM KCl, 0.1 mM CaCl_2_, 5 mM MES, 2 mM Tris base, pH 6.0) supplemented or not with 150 mM NaCl. After 60 min of treatment, control, and NaCl treated seedlings were washed with distilled water and a solution of 0.5 mM CaCl_2_. Root segments (1–1.5 cm long) were cut from the mature zone to 1–2 cm from the root apex and were incubated for 60 min in Eppendorf tubes of 500 μL (measurement solution) with 10 μM of Sodium Green™ (sodium staining) and 20 μM of FM^®^4-64 (membrane staining, T-13320, Molecular Probes, Life Technologies). Excess dye was removed, and the primary root segments were placed on a slide. Approximately 10 μL of Vectashield (H-1000, Vector Laboratories, Inc.) with DAPI (nuclei staining, D3571, Molecular Probes, Life Technologies) was added. The fluorescence was observed using the confocal microscope FluoView™ FV1000 (Olympus, Japan). A UPLFLN 40X0 (oil, NA: 1.3) lens was used with a scanning speed of 10 μs/pixel. DAPI, Sodium Green™, and FM4-64 exhibit excitation and emission wavelengths of 358–461 nm, 507–532 nm, and 515–670 nm, respectively. Z images had a resolution of 512 × 512 pixels and were projected as a single image. Fluorophores were merged using the software FV10-ASW 3.01b.

### Statistical analysis

Data were analyzed using a One-Way analysis of variance (ANOVA) (Sigma Stat Version 3.1). Treatment averages were compared using Tukey's range test.

## Results

### Varieties of *C. chinense* differ in sensitivity to NaCl stress

**Figure 1:**
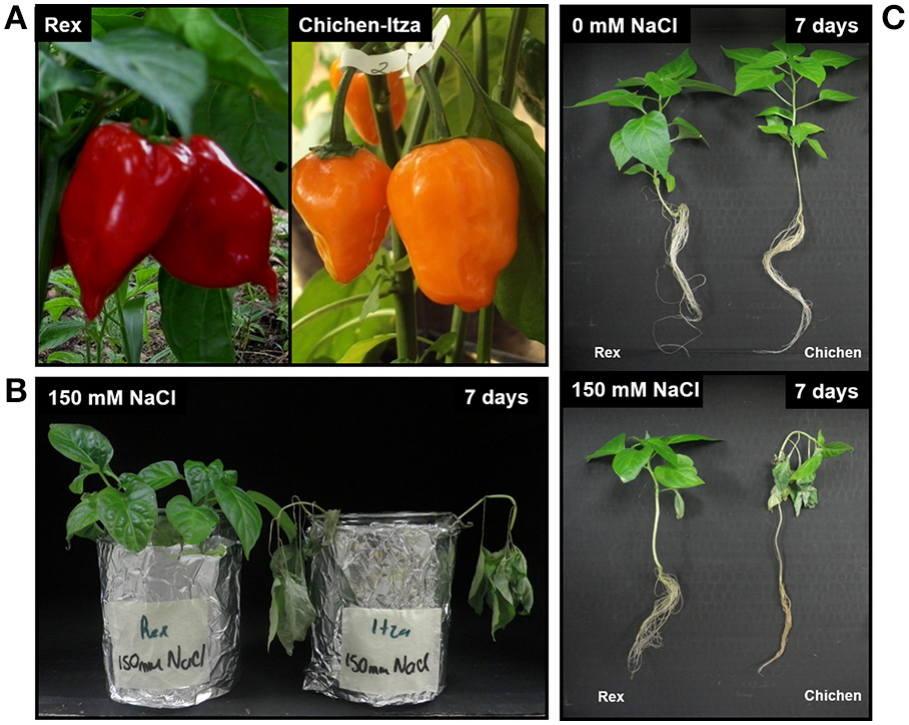
**Morphologic and sensitivity differences to NaCl stress in two varieties of *C. chinense*. (A)** Representative color of ripe habanero pepper fruits of the Rex (red) and Chichen-Itza (orange) varieties. **(B)** Forty-five-day-old seedlings of two varieties of *C. chinense* after 7 days of culture in H1/5 solution supplemented with 150 mM NaCl. **(C)** Appearance and sensitivity of Rex and Chichen-Itza seedlings after 7 days of treatment under control conditions (top) and 150 mM NaCl (bottom).

Different varieties of *C. chinense* such as Rex and Chichen-Itza differ in their morphologic characteristics (fruit color, size, and root system architecture), as shown in Figures 1A,C. These two varieties exhibit differing sensitivities to salt stress, Rex being more tolerant than Chichen-Itza (Figures 1B,C). A concentration of 150 mM of NaCl over 7 days of culture in hydroponic conditions had a strong impact on the growth of the two varieties. Loss of turgor, leaf abscission, and darkening of the root system were observed, especially in the variety Chichen-Itza (Figures 1B,C).

**Figure 2:**
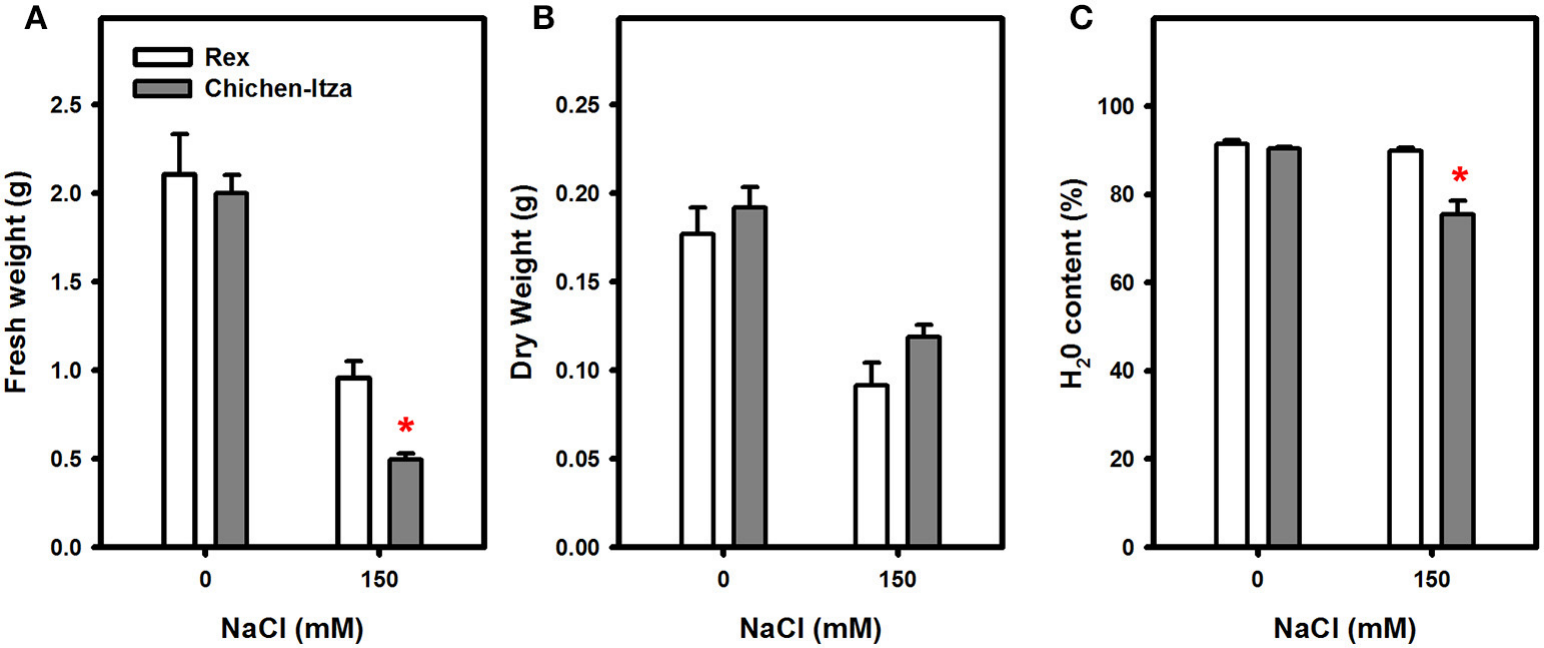
**Effect of saline stress (150 mM NaCl) on fresh weight (A), dry weight (B), and water content (C) of Chichen-Itza and Rex varieties seedlings after 7 days of culture with 150 mM NaCl**. The white bars (Rex) and gray bars (Chichen-Itza) represent the mean FW, DW, and WC for the different treatments, ME ± SD (*n* = 6 seedlings). The asterisk indicates statistically significant differences between varieties for each treatment (*P* < 0.050, Tukey's test).

A significant reduction of fresh and DWs was also induced by NaCl in both genotypes (Figures 2A,B). Under salt stress, the FW reduction was greater in Chichen-Itza, (75%) than in the Rex variety (50%) (Figure 2A). The water content in the Rex variety was identical to the water content in control seedlings with no salt treatments. However, the water content in the Chichen-Itza variety was significantly lower than in the untreated controls (Figure 2C). It is noteworthy that although symptoms of stress (wilting and senescence) were observed at concentrations below 150 mM NaCl, the effect on growth parameters was not significant between varieties by the end of the measurement period (data not shown). For this reason, a dose of 150 mM NaCl was selected for subsequent studies. This concentration has been used in various studies with glycophytes such as *A. thaliana*, *S. tuberosum*, and *S. lycopersicum* (Apse et al., 1999; Rodríguez-Rosales et al., 2008; Jaarsma et al., 2013).

### Effect of NaCl on proline accumulation in different varieties of *C. chinense*

**Figure 3:**
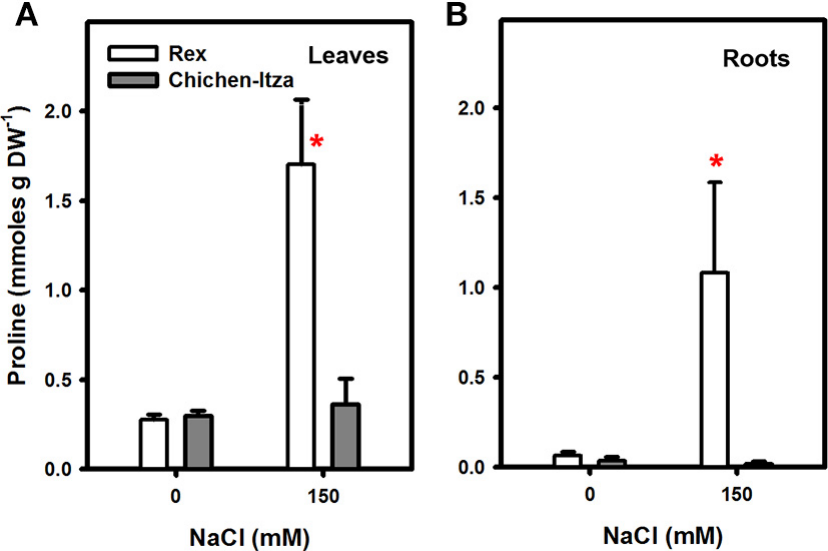
**Effect of NaCl treatment on proline accumulation in leaves (A) and roots (B) of two varieties of *C. chinense***. The bars (white: Rex, gray: Chichen-Itza) represent the means of treatments with or without NaCl (150 mM), ME ± SD (*n* = 3). The asterisk indicates statistically significant differences between varieties for each treatment (*P* < 0.050, Tukey's test).

Many species of plants accumulate compatible solutes, such as proline, in response to abiotic stresses such as drought and salinity. In the varieties of *C. chinense* studied, proline accumulation was analyzed in the leaves and roots of seedlings grown in hydroponic cultures and subjected to 0 (control) or 150 mM NaCl for 7 days. Proline concentrations in the roots and leaves were similar in both varieties in the absence of salt. The proline concentration was higher in the leaves than in the roots (Figure 3). After 7 days of salt stress, the proline content in the Rex variety increased approximately 6.0-fold compared to control. However, in the Chichen-Itza variety, the values were similar to those in control seedlings growing without NaCl (Figure 3A). The same effect was observed in the roots as in the leaves. In the Rex variety, proline levels were 16-fold higher compared to control (Figure 3B). Surprisingly, the accumulation of proline in the roots was 50-fold higher in the Rex than in the Chichen-Itza variety after 7 days of treatment with NaCl (Figure 3B).

### Potassium retention and sodium accumulation in roots under salt stress

**Figure 4:**
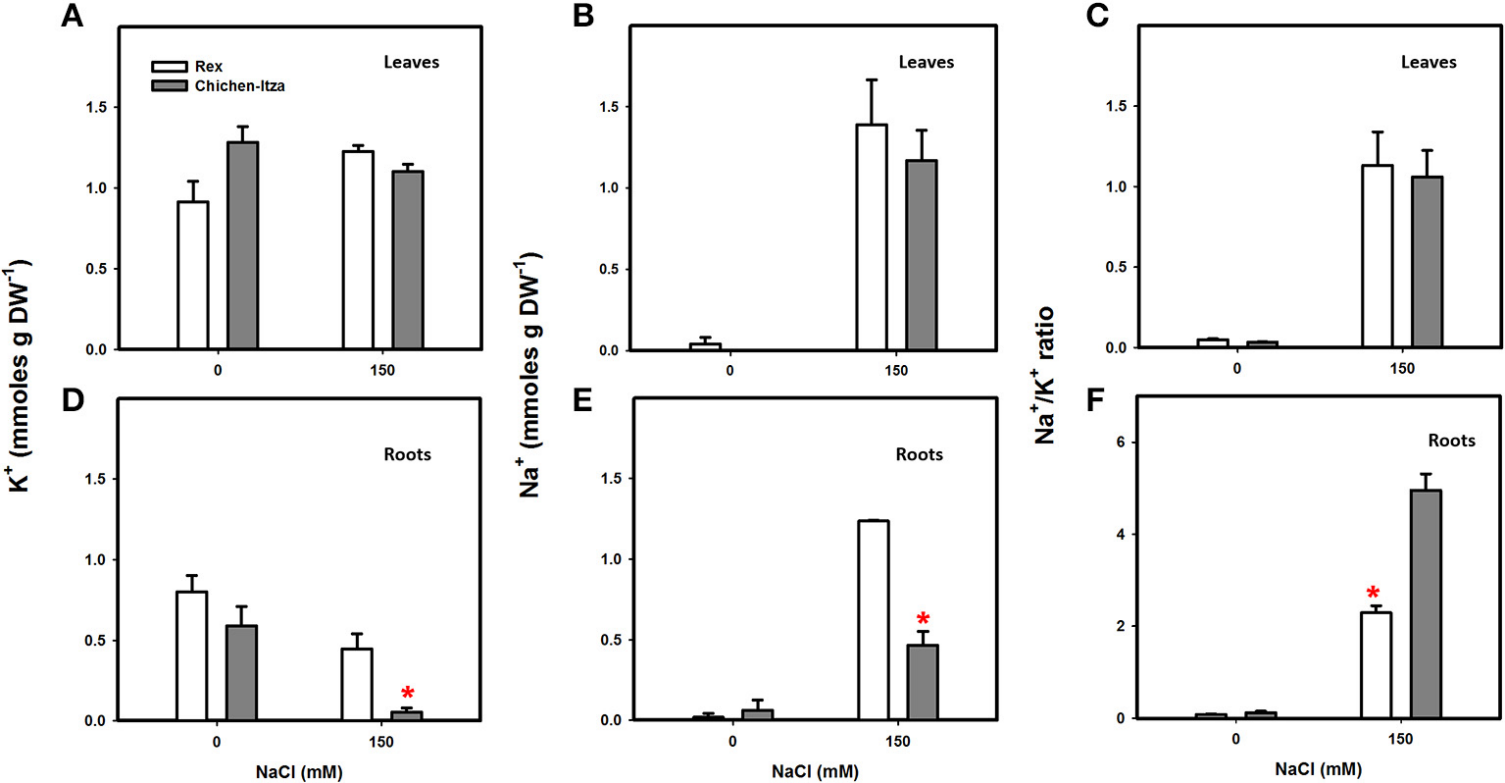
**Potassium and sodium content in two varieties of *C. chinense* under NaCl stress conditions**. Forty-five-day-old seedlings cultivated in hydroponic cultures (H1/5) for 7 days with 0 and 150 mM of NaCl. K^+^ content in the leaves **(A)** and roots **(D)** of Rex and Chichen-Itza varieties after treatment with salt. Na^+^ content in the leaves **(B)** and roots **(E)** of the two *C. chinense* varieties after 7 days of treatment. The Na^+^/K^+^ ratios in leaves **(C)** and roots **(F)** in control and NaCl treatments. Bars represent the average of the treatments with or without NaCl (150 mM), ME ± SD (*n* = 3). The asterisk indicates statistically significant differences between varieties for each treatment (*P* < 0.050, Tukey's test).

K^+^ and Na^+^ accumulation patterns in the two varieties of *C. chinense* are presented in Figure 4. In the absence of salt stress, K^+^ content in the leaves and roots did not significantly differ between these varieties (Figures 4A,D). A higher K^+^ concentration was observed in the leaves as compared to roots, due to the K^+^ accumulation on the top of transpiration stream (Conn and Gilliham, 2010). NaCl stress did not modify K^+^ content in the leaves in either variety of *C. chinense* (Figure 4A). However, a significant decrease in K^+^ was observed in the roots of seedlings from the Chichen-Itza variety under salt stress. No significant reduction of root K^+^ was observed in the Rex variety (Figure 4D). Furthermore, no differences in K^+^ were observed for concentrations lower than 150 mM of NaCl in either variety (Figure S1).

In absence of salt stress, Na^+^ content in the leaves and roots of both varieties was minimal, with values of 0.02 mmol and 0.04 mmol g DW^−1^, respectively. Treatment with 150 mM NaCl increased the Na^+^ content in the leaves; however, no differences were observed between the two varieties (Figure 4B). Na^+^ content increased in the roots treated with salt in both varieties and was surprisingly higher in Rex (twofold) compared to Chichen-Itza variety (Figure 4E). The Na^+^/K^+^ ratio in leaves and roots were very similar between pepper varieties when NaCl was not supplied (Figures 4C,F). As a consequence of increase in Na^+^ and decreases in K^+^ content by NaCl treatment, Chichen-Itza variety exhibited much higher Na^+^/K^+^ ratio in roots compared to the Rex variety (Figure 4F). In contrast, the Na^+^/K^+^ ratio in leaves at NaCl treatment did not differ between the genotypes (Figure 4C). Furthermore, at low and moderate concentrations of NaCl, the Rex variety exhibited higher Na^+^ content in the roots and much lower Na^+^ content in the leaves. The opposite effect was observed in the Chichen-Itza variety at 50 mM NaCl. Similar Na^+^ content was observed between shoots and roots at a concentration of 100 mM (Figure S2).

### NaCl induces K^+^ efflux in roots of *C. chinense*

**Figure 5:**
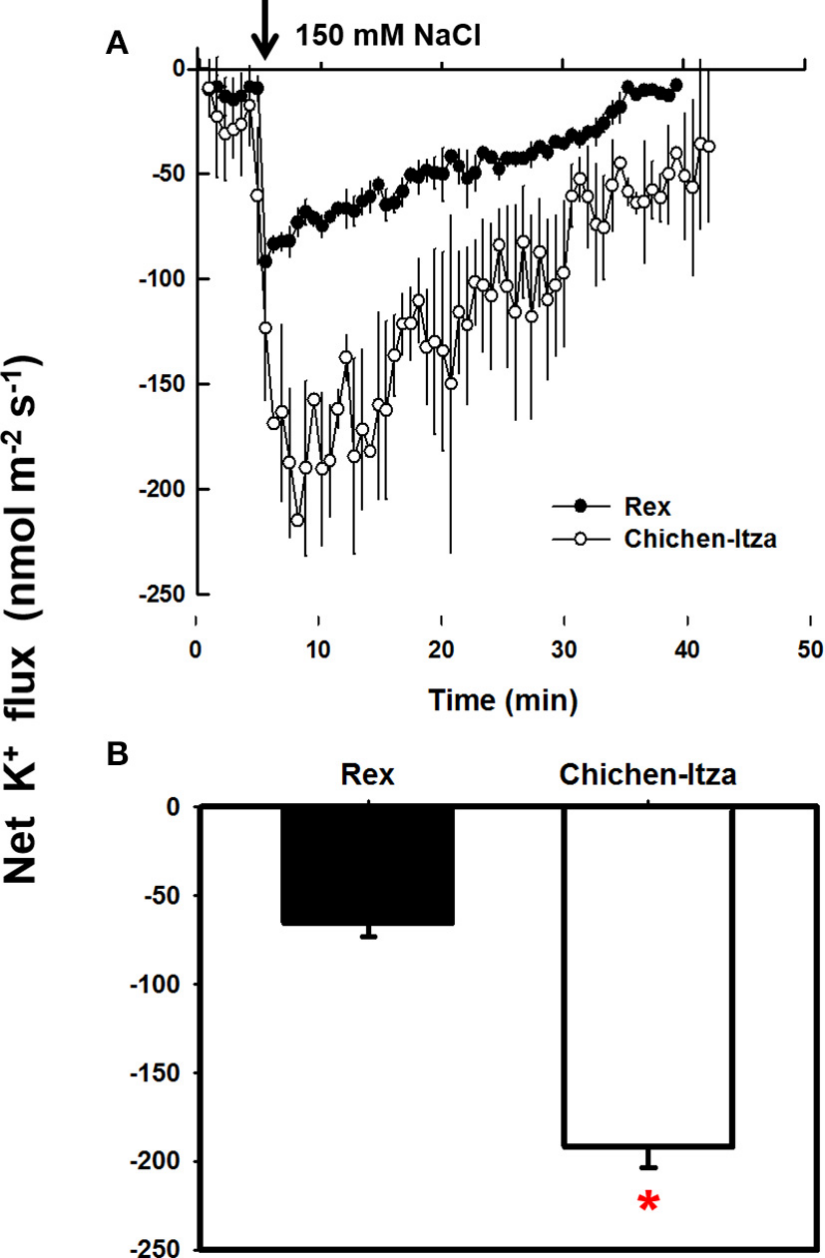
**Measurement of the net flow of K^+^ in the roots of *C. chinense* in response to NaCl stress. (A)** Kinetics of net K^+^ flux measured in the mature root zone of seedlings (10 days old) of the Rex (closed circles) and Chichen-Itza (open circles) varieties in response to 150 mM NaCl (the arrow indicates the time of addition of the treatment). **(B)** Mean net flow of K^+^ from the root of each variety in the first 10 min after application of 150 mM NaCl. ME ± SD (*n* = 4–5). The asterisk indicates statistically significant differences between varieties by treatment (*P* < 0.001, Tukey's test).

As described so far, Chichen-Itza and Rex varieties differ in their sensitivity to salt stress, the latter being less affected (Figures 1, 2). The Rex variety retains 55% of K^+^ in salt-stress conditions, whereas Chichen-Itza looses about 90% of the root K^+^ (Figure 4). To deepen the study of this response, the K^+^ flux was measured using the non-invasive MIFE technique. The addition of 150 mM NaCl induced K^+^ efflux from the epidermal cells in the mature root zone of the two varieties of *C. chinense* (Figure 5). This efflux started immediately following NaCl treatment. A higher K^+^ efflux was observed in the roots of the Chichen-Itza variety compared to Rex variety (Figure 5A). The difference in K^+^ efflux in the 1 min was double and the relative difference even increased with time (Figure 5B). In the Rex variety K^+^ efflux was close zero after 35 min, whereas in Chichen-Itza a significant K^+^ efflux of about 50 nmol m^−2^ s^−1^ was observed at late times.

### Effect of NaCl on H^+^ efflux in roots of *C. chinense*

**Figure 6:**
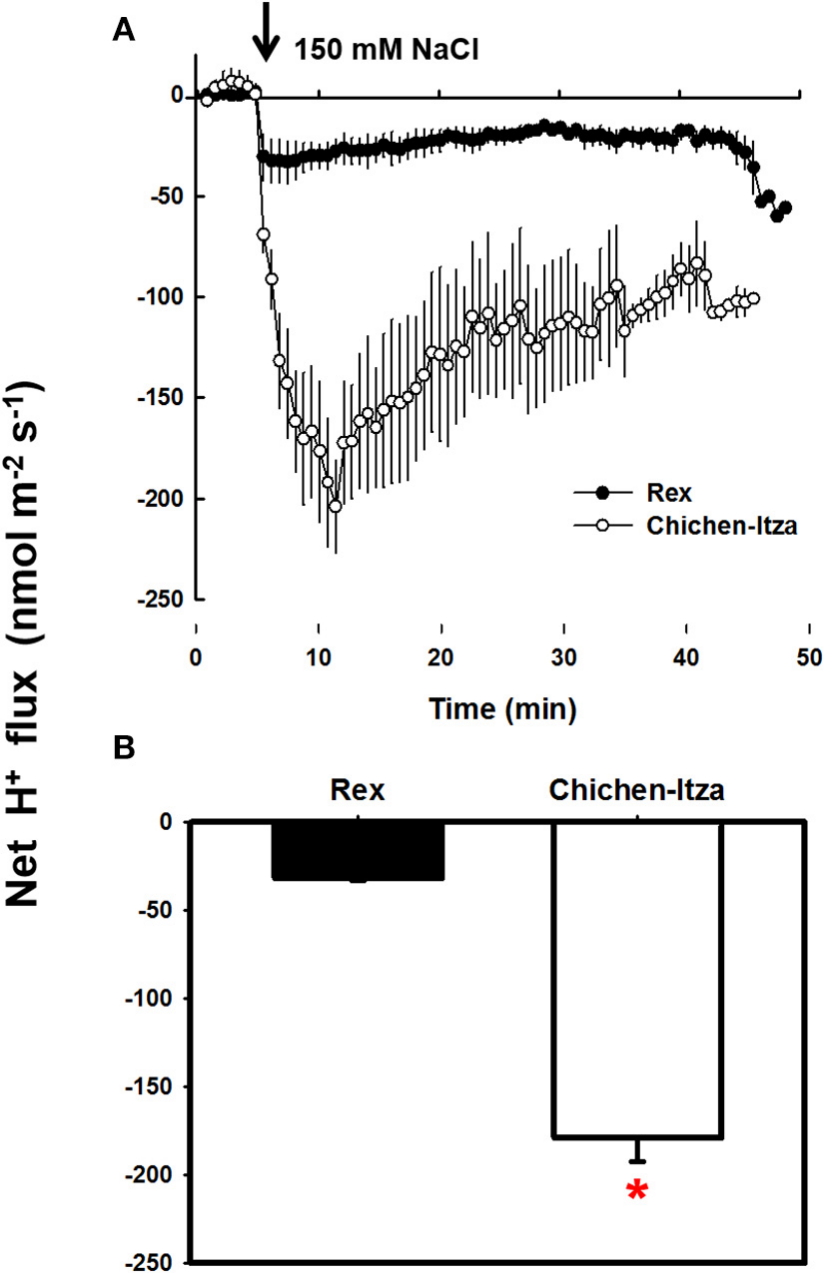
**Effect of NaCl on the net H^+^ flux in the mature root area of *C. chinense*. (A)** Kinetics of net flux of H^+^ measured in the roots of 10-day-old seedlings of Rex (closed circles) and Chichen-Itza (open circles) varieties after adding 150 mM NaCl (arrow indicates the time addition of the treatment). **(B)** Mean net flow of H^+^ from the root of each variety within the first 10 min of application of 150 mM NaCl. ME ± SD (*n* = 4–5). The asterisk indicates statistically significant differences between varieties by treatment (*P* < 0.001, Tukey's test).

**Figure 7:**
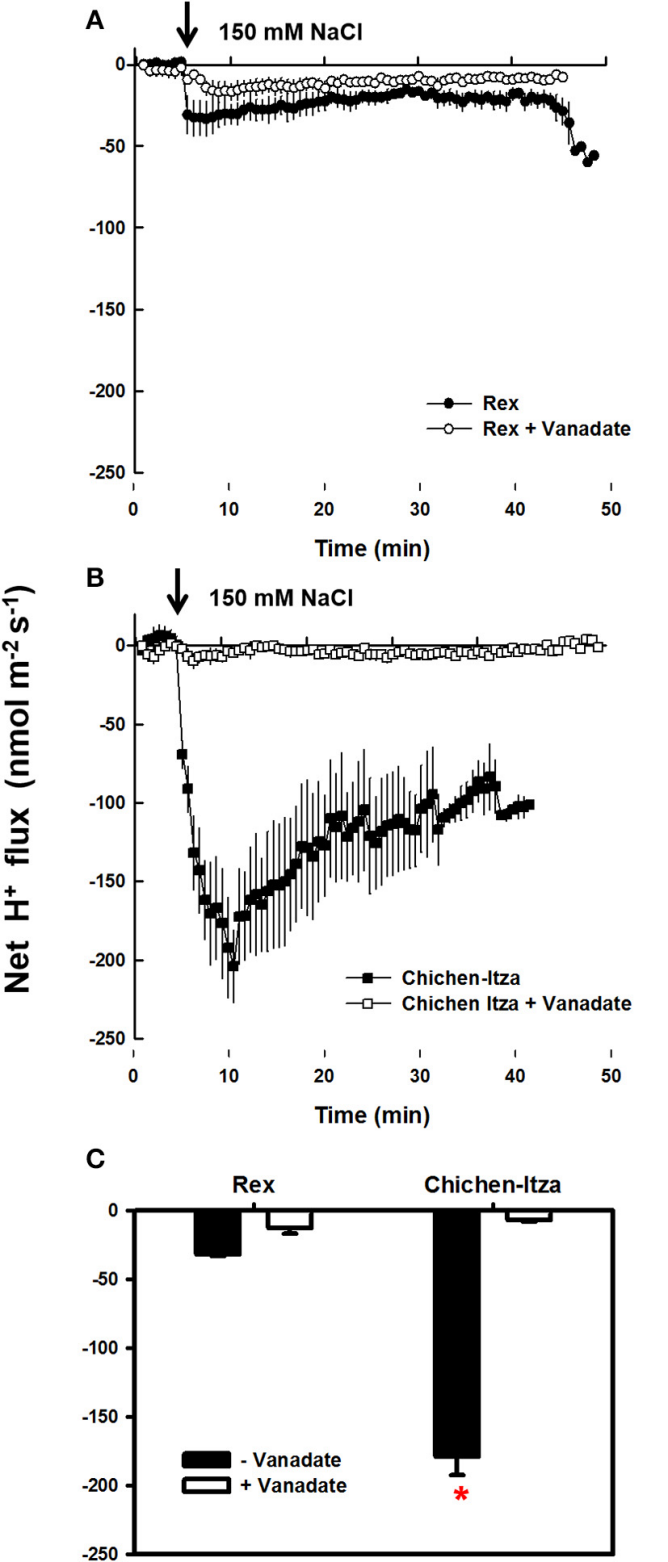
**Effect of vanadate pre-treatment on NaCl-induced H^+^ efflux in roots of *C. chinense*. (A)** Kinetics of net H^+^ flux measured in the mature root zone of the Rex variety and **(B)** of the Chichen-Itza variety. 10-day-old roots of the two strains were pre-treated with 1 mM vanadate for 45–60 min before adding of 150 mM NaCl (marked by an arrow). Open circles and squares indicate pre-treatment with vanadate. Closed circles and squares indicate no pre-treatment. **(C)** Average effect of vanadate pre-treatment on the net flow of H^+^ from the root of each variety in the first 10 min after the application of 150 mM NaCl, ME ± SD (*n* = 3–4). The asterisk indicates statistically significant differences between varieties by treatment (*P* < 0.001, Tukey's test).

In the roots of habanero pepper, NaCl stress caused significant changes in the net flux of H^+^ (Figure 6). Before starting the salt treatment (first 5 min), the net flux of H^+^ was zero in both varieties. Application of 150 mM NaCl induced a substantial H^+^ efflux (Figure 6A). In the roots of the Rex variety, NaCl induced H^+^ efflux was much lower as compared the Chichen-Itza variety (Figure 6B). Furthermore, a pre-treatment of seedlings with 1 mM sodium orthovanadate (an inhibitor of P-type H^+^-ATPases) strongly suppressed the H^+^ efflux from the mature root zone of both genotypes (Figure 7). Thus, the NaCl- induced H^+^ efflux was mediated by P-type H^+^-ATPases.

### Na^+^ subcellular localization in roots

**Figure 8:**
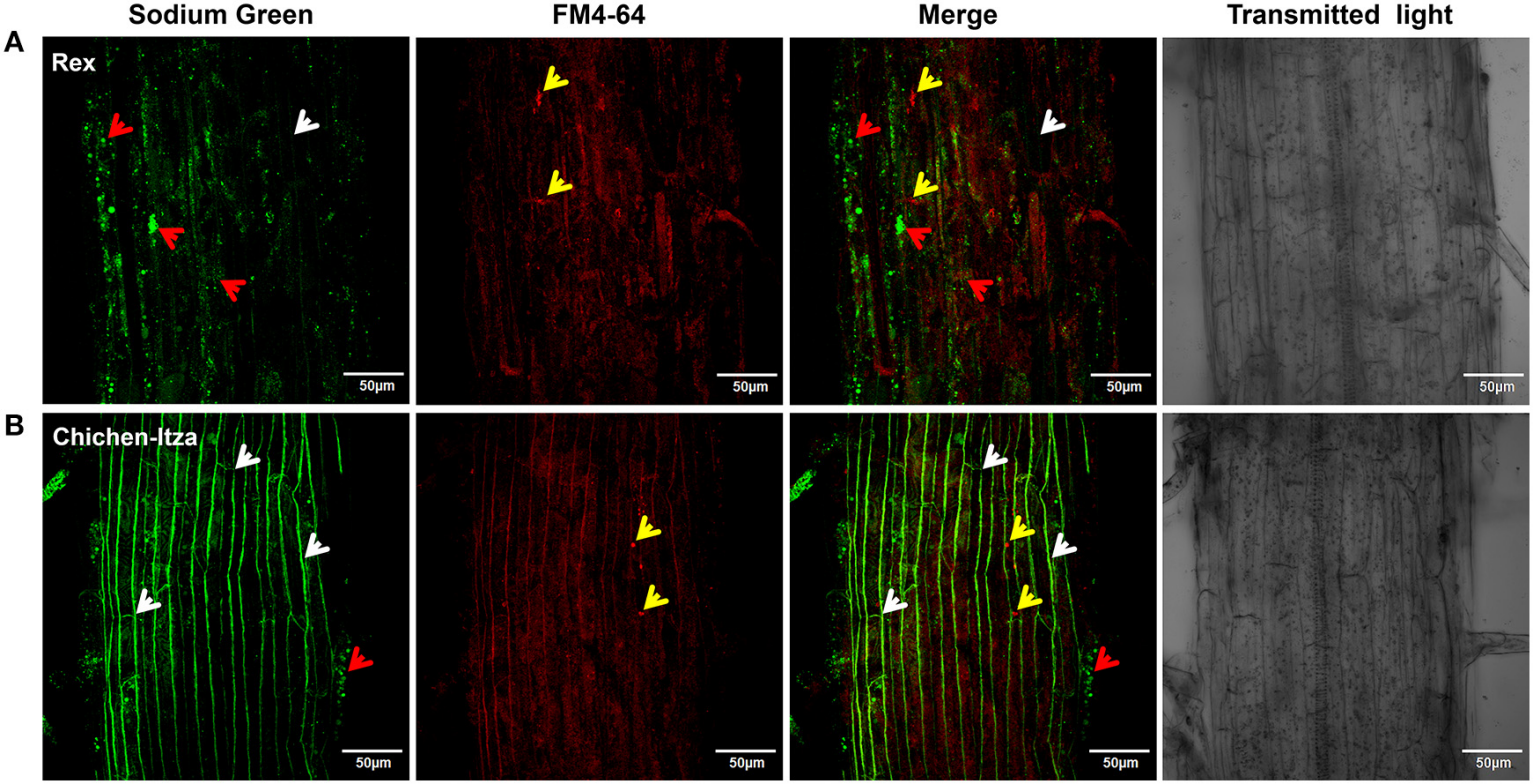
**Sodium localization in epidermal cells of the primary root of habanero pepper *C. chinense*). (A)** Localization of Na^+^ in the roots of the Rex and **(B)** Chichen-Itza varieties. 10-day-old habanero pepper roots were treated with 150 mM NaCl for 60 min and then stained with Sodium Green (Na^+^ detection) and FM4-64 (membrane staining) before the confocal images were taken. White arrowheads indicate the location of the Na^+^ around cells. Red arrowheads indicate the intracellular localization of Na^+^, and yellow arrowheads indicate the presence of endosomes. Fluorescence analysis was determined in the mature root zone. Images are representative of the analysis of three roots per treatment and variety.

**Figure 9:**
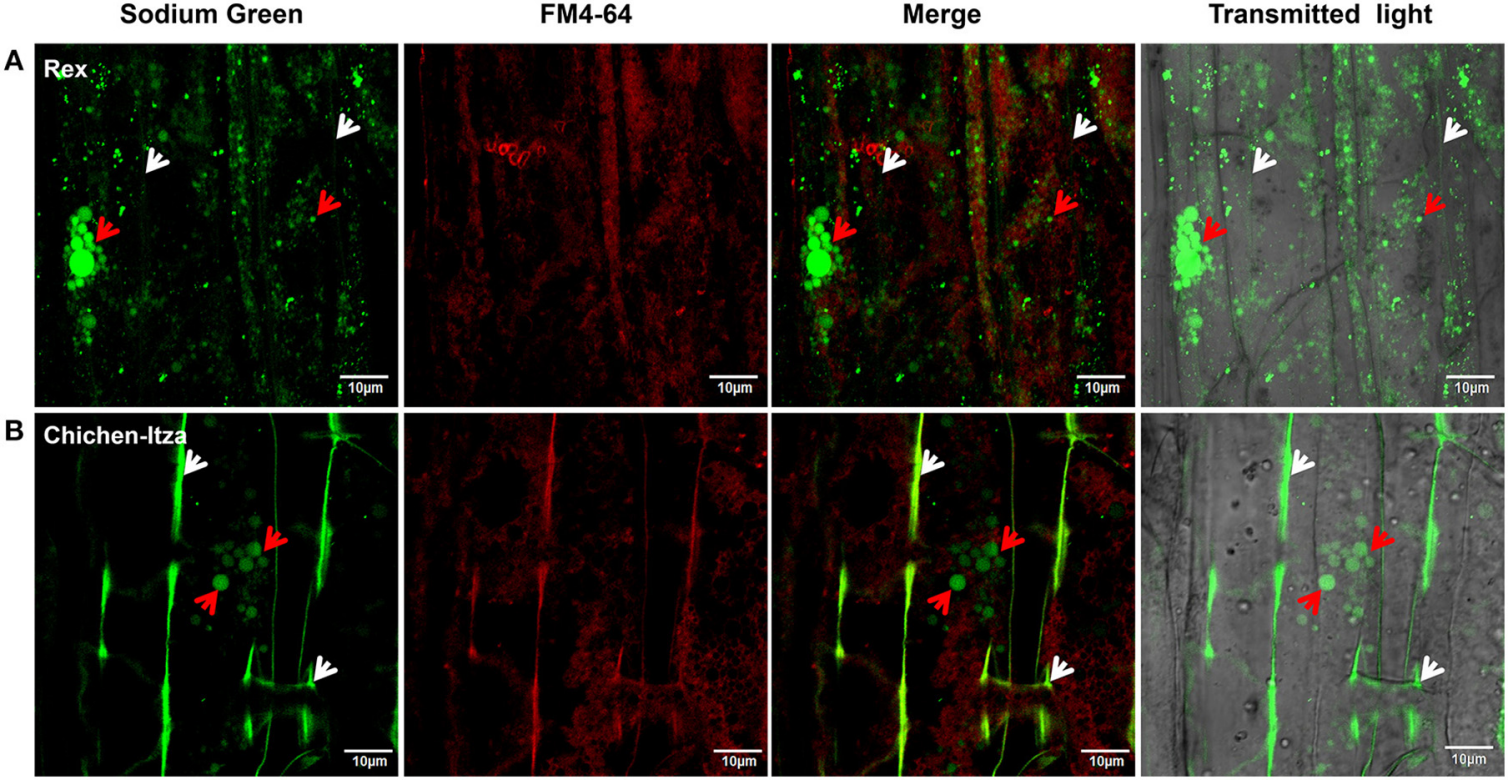
**Sodium subcellular localization in the apoplast and subcellular compartments**. Magnified images of the mature root area shown in Figure 8. **(A)** Localization of Na^+^ in the roots of the Rex and **(B)** Chichen-Itza varieties. The white arrowheads indicate the location of Na^+^ in the apoplast, and red arrowheads indicate the location of Na^+^ in subcellular structures. Images are representative of the analysis of three roots per treatment and variety.

After 60 min of treatment with 150 mM NaCl, marked differences were observed with respect to Na^+^ localization in the mature root zone between two pepper genotypes (Figure 8). In the Rex variety, Sodium Green™ fluorescence was observed in vacuole-like structures of epidermal cells (red arrowheads, Figure 8A), suggesting an efficient mechanism for sodium compartmentalization in this variety. By contrast, in the Chichen-Itza variety, green fluorescence was observed around the epidermal cells in the mature root zone (white arrowheads, Figure 8B). Small endosomes stained with FM4-64 dye were observed in both pepper varieties under salt stress (yellow arrowheads, Figure 8). However, green fluorescence was not evident in these structures, indicating the absence of Na^+^ accumulation (merge, Figure 8). In epidermal cells of the Rex variety, a conglomeration of small structures with green fluorescence was observed around a larger structure comparable to a vacuole (red arrowheads, Figure 9A); similar structures were previously observed in *Arabidopsis* (Hamaji et al., 2009). In contrast to the Rex variety, in the Chichen-Itza a less pronounced and diffuse pattern of small compartments stained with Sodium Green™ was observed (red arrowheads, Figure 9A). Overall, the highest levels of fluorescence were observed outside the cells in Chichen-Itza (white arrowheads, Figure 9B), demonstrating that Na^+^ was mainly located in the apoplast. In control roots, Na^+^ indicator did not report any change of fluorescence for either variety (Figure S3).

## Discussion

### Sensitivity and growth in *C. chinense* seedlings under NaCl stress

Most crop plants that provide food for the world population are glycophytes and are very sensitive to high concentrations of salts in the soil, mainly NaCl. Salt stress is the main abiotic factor that affects growth, yield, and quality. Peppers (*Capsicum* spp.) are a major vegetable crop and are not exempt from the effect of salt throughout their ontogeny (Bojórquez-Quintal et al., 2012). Notably, pepper plants differ in their sensitivity to salt stress, including marked differences between varieties within the same species (Aktas et al., 2006). In this work, two varieties of *C. chinense* Jacq. were used as models. *C. chinense* Jacq. is a species in high demand in southeastern Mexico for its flavor and pungency and is commonly known as habanero pepper. These varieties exhibit different morphological characteristics and were shown to differ in their sensitivity to salt stress, with the Rex variety being more tolerant than the Chichen-Itza variety (Figure 1).

NaCl concentrations between 0 and 150 mM affect the growth of pepper plants, depending on the genotype, species, and condition of growth (Bojórquez-Quintal et al., 2012). In this study, the application of 150 mM NaCl had a dramatic impact on the growth of both varieties of *C. chinense.* Similar results were reported in *C. annuum* (Aktas et al., 2006). The FW, turgor, and water content of the Rex variety were less affected (Figure 2). In this variety, 70% of seedlings survived after treatment with NaCl, compared with 10% of the Chichen-Itza variety (data not shown). These results suggest the existence of intrinsic mechanisms of tolerance in the Rex variety to avoid the deleterious effect of NaCl.

### Differences in proline accumulation between varieties of habanero peppers

Proline accumulation is one of the most common and important responses of plants to adverse environmental factors such as drought and salt stress. Proline is a multifunctional amino acid participating in a wide range of functions (Szabados and Savouré, 2009) and represents a potential biochemical marker for the salt tolerance in plants (Ashraf and Harris, 2004). In our study, different proline content was observed in two pepper varieties, contrasting in their salt sensitivity (Figure 3). In leaves of the Rex variety, proline content increased six times in the presence of NaCl, as compared to a non-significant increase in Chichen-Itza leaves. The leaves are the major site of proline synthesis (source organ). It has been suggested that proline accumulation in this organ occurs to maintain chlorophyll content and turgor to protect the photosynthetic activity under salt stress conditions (Yildiztugay et al., 2011; Huang et al., 2013). Furthermore, treatment with NaCl induced a dramatic (16-fold) increase in proline content in the roots, but only of the tolerant variety Rex. At low water potential, proline is thought to be transported from the leaf (source) to the roots (sink) for growth processes or other functions, such as osmotic adjustment depending on proline content (Sharma et al., 2011). Exogenous administration of proline reduced NaCl- induced K^+^ efflux in barley and Arabidopsis roots (Cuin and Shabala, 2005, 2007a,b). In *Solanaceae* such as the potato (*S. tuberosum*), increased proline content correlates with a higher expression of *P5CS* (synthesis gene) and decreased *PDH* gene expression (degradation) in tolerant but not in sensitive cultures (Jaarsma et al., 2013). Furthermore, overexpression of *P5CS* in *N. tabacum* and *S. tuberosum* stimulated proline accumulation under NaCl stress and improved the tolerance (Kishore et al., 1995; Hmida-Sayari et al., 2005). However, there is not always a good correlation between the accumulation of this osmolyte and tolerance to salt stress, and whether it is a symptom of damage or an indicator of tolerance is a matter of debates. For example, in rice (*O. sativa*), soybean (*G. max*), tomato (*S. lycopersicum*), and barley (*H. vulgare*), a negative correlation between proline accumulation and tolerance to stress has been reported. In these studies, sensitive genotypes accumulated more proline (Moftah and Michel, 1987; Aziz et al., 1998; Lutts et al., 1999; Chen et al., 2007a). In addition, proline synthesis is a metabolically expensive strategy (Shabala and Cuin, 2007; Shabala and Shabala, 2011). Nevertheless, proline content has been shown to be higher in many plant varieties tolerant to salt relative to their susceptible counterparts (Ashraf and Harris, 2004), as also demonstrated by the results of this work (Figure 3).

### The tolerant pepper variety accumulates more sodium in the roots than the sensitive variety

The hyperaccumulation of Na^+^, particularly in the leaves, inhibits protein synthesis, enzymatic activity, and photosynthesis. Therefore, plants have the ability to control the transport and distribution of Na^+^ to organs, tissues and cells where it causes less damage to protect against the accumulation of this cation. The most sensitive glycophytes (cereals or vegetables) are unable to control the transport of Na^+^; therefore, large amounts of this ion are translocated to the shoot (Maathuis, 2014), inducing senescence, growth inhibition, and eventually death of the plant (Roy et al., 2014). In contrast, most halophyte and some glycophytes plants tend to accumulate large amounts of Na^+^ in the leaves. It has been suggested that these plants use Na^+^ in addition to K^+^ to maintain turgor and growth (Hariadi et al., 2011; Adolf et al., 2013; Bonales-Alatorre et al., 2013a,b; Maathuis, 2014).

In our study (Figure 4), we observed that two varieties of habanero pepper (*C. chinense*) exhibit the same Na^+^ content in the leaves but exhibit differences in Na^+^ accumulation in the roots (after a minimum of 7 days of exposure to 150 mM NaCl). In plants of *C. annuum* treated with NaCl, a higher Na^+^ content has been reported in the shoots of sensitive genotype*s* compared to tolerant genotypes. In fact, a positive correlation was observed between the severity of the symptoms in the leaves and Na^+^ concentrations in the shoots (Aktas et al., 2006). This suggests an effect dependent on the accumulation of Na^+^ as proposed Roy et al. (2014). The senescence and the severity of the symptoms observed in the aerial part of *Capsicum chinense* in this study (Figure 1) seem to be due to an osmotic effect rather than an ionic effect due to the presence of Na^+^ in leaves (Figure 4B). In addition, this osmotic effect can be avoided by the accumulation of proline in the Rex variety (Figure 3A) as described in the previous section, as the effect of NaCl was less severe in that variety.

The root system is the first site of detection and the first line of defense against excess Na^+^ in cells (Ji et al., 2013). NSCC are the principal source of Na^+^ influx in the plant cell (Demidchik and Tester, 2002). As suggested for other species (Demidchik and Maathuis, 2007), NSCC can mediate Na^+^ influx of pepper plants (Rubio et al., 2003). In our study, the Rex variety, less affected by salt, exhibited a two–fold higher Na^+^ content in the root than in the Chichen-Itza variety (Figure 4E). Furthermore, the accumulation of Na^+^ in the root system of the tolerant variety was higher at low NaCl concentrations (50 mM) where reached its peak and was maintained at higher NaCl concentrations (150 mM). The levels of Na^+^ in the roots of the Chichen-Itza variety were lower and stable at all of the NaCl concentrations tested (Figure S2). Overall, the accumulation of Na^+^ is higher (in both roots and leaves) in the tolerant variety than in the sensitive variety (Figure S1 and Figure 4). At low and moderate salt concentration ranges, the Rex variety (tolerant) possesses more Na^+^ in the roots and much less in the leaves. However, the opposite was observed in the Chichen-Itza variety (sensitive) at 50 mM NaCl. Furthermore, at 100 mM NaCl, the same Na^+^ content was observed between the shoots and roots in the Chichen-Itza variety (Figure S2). The higher Na^+^ content in roots than in leaves suggests that exclusion mechanisms (SOS1, antiporters, and HKT1 transporters) and compartmentalization (NHX, antiporters) are present in roots and efficiently reduce the Na^+^ transport to leaves. These mechanisms have also been reported in other members of *Solanaceae*, such as tomatoes and potatoes (Queirós et al., 2009; Rodríguez-Rosales et al., 2009; Almeida et al., 2014). In particular, the HKTs (subfamily 1) exhibit an important role in the recovery of Na^+^ from the xylem to prevent its transport to the aerial part, and recirculate Na^+^ to the roots (Horie et al., 2009; Almeida et al., 2013; Adams and Shin, 2014).

### Na^+^ subcelular localization in the roots: compartmentalization and Na^+^ efflux in different varieties of *C. chinense*

After 60 min of treatment with 150 mM NaCl, Na^+^ was mainly located in vacuole-like structures in root epidermal cells in the Rex variety (Figure 9). This result suggests the existence of an efficient mechanism for Na^+^ compartmentalization in this genotype. Similarly, it has been reported that Na^+^ is confined in epidermal cells vacuoles and in the cortex in the roots of *Arabidopsis* and *Citrus* (Oh et al., 2009; Gonzalez et al., 2012). Furthermore, it is noteworthy that in the two varieties of habanero pepper, small compartments were stained with the fluorophore. These compartments were found in greater quantities in the roots of the Rex variety (Figure 9). Similar results were observed in the roots of *A. thaliana* under salt stress. Hamaji et al. (2009) reported that Na^+^ accumulates in the vacuoles as well as in small vesicular compartments around vacuoles. These authors suggest that the fusion of these vesicles with the main vacuole increases its size and the tolerance to excess salt. This explanation is logical, as we observed a conglomeration of structures stained with the fluorophore in the roots of the tolerant variety after a short period of NaCl stress (Figure 9). In tolerant salt includes such as mangroves and barley, a rapid increase in vacuolar volume in response to salt stress was observed. This phenomenon was not reported in sensitive species such as peas and tomatoes (Mimura et al., 2003).

Recently, in tobacco salt-acclimated BY2 cells accumulation of Na^+^ in vacuoles and small vesicles was reported. Interestingly, the putative VAMP711 (vesicle-associated membrane protein 711) and VPS46 (charged multivesicular body protein) proteins were highly induced in this BY2 cells, suggesting a Na^+^ transport mechanism for vesicle trafficking (Garcia de la Garma et al., 2014). Furthermore, the increased Na^+^ sequestration by vacuolar and small compartments in the Rex variety could be due to increased expression of NHX transporters as observed in tomato species with different sensitivity to salt (Galvez et al., 2012). Also, the up-regulation of V-ATPase (vacuolar-type H^+^-ATPase) and H^+^-PPase (vacuolar H^+^-pyrophosphatase) in Rex variety may result in a higher proton electrochemical gradient, which facilitates enhanced sequestering of ions into the vacuole and endosomes, reducing water potential and resulting in increased salt tolerance (Gaxiola et al., 2001; Bassil et al., 2012; Pittman, 2012). In different species of plants, overexpression of AVP1 (vacuolar H^+^-PPase) and co-overexpression with AtNHX1 enhances salt tolerance (Gaxiola et al., 2001; Pasapula et al., 2011; Shen et al., 2014). Finally, the reduction of Na^+^ loss via non-selective vacuolar channels could assist efficient vacuolar Na^+^ accumulation (Bonales-Alatorre et al., 2013b). In the Rex variety, all or some of these mechanisms may be involved in the Na^+^ compartmentalization.

On the contrary, in the Chichen-Itza variety, Na^+^ was mostly observed in the apoplastic area between cells of the root (Figures 8, 9). This result suggests an intensive Na^+^ extrusion toward the outside of the cell, likely via the Na^+^/H^+^ antiporters (SOS1) of the plasma membrane (Shi et al., 2002). This is consistent with a low content of Na^+^ (Figure 4) and with the large active H^+^ efflux, mediated by vanadate-sensitive H^+^-ATPase, in the roots of this variety. However, the high rate of Na^+^ extrusion in sensitive cultivars could have a high energetic cost (Malagoli et al., 2008). This opts for the use of intracellular Na^+^/H^+^ (NHX-type) antiporters as compared to SOS1-type ones; the latter may be more useful in early responses to acute salt stress. Candidate genes for the intracellular (NHX) and plasma membrane (SOS1) exchangers were revealed in the genome of *Capsicum annuum* (Qin et al., 2014).

Furthermore, we may not exclude at this moment that accumulation of the indicator at cell walls, which possess high esterase activity, could contribute to the observed fluorescence signal and the difference between Rex and Chichen-Itza.

### K^+^ retention in the roots is a tolerance mechanism in habanero pepper plants

Potassium is an essential nutrient throughout the life cycle of plants, including the adaptation to hostile environments. The regulation of K^+^ homeostasis plays a central role in tolerance to biotic and abiotic stresses in plants (Anschütz et al., 2014; Demidchik, 2014; Shabala and Pottosin, 2014). K^+^ efflux from the root is a common physiological reaction that occurs under a wide range of stress conditions (Demidchik, 2014). K^+^ retention in roots has been proved to confer salt tolerance in barley, wheat, lucerne, and poplar (Chen et al., 2007b,c; Cuin et al., 2008; Smethurst et al., 2008; Sun et al., 2009). In this study, the treatment with 150 mM NaCl significantly decreased the content of K^+^ in the roots of the sensitive variety but not in the tolerant variety of habanero pepper (Figure 4D). In contrast, K^+^ content in the leaves was not affected by treatment with NaCl in either genotype (Figure 4A). These data suggest that the ability to retain K^+^ by roots is one of the salt tolerance mechanisms of *C. chinense.*

Initial NaCl induced K^+^ efflux was higher in the Chichen-Itza as compared to the Rex variety and doubled after 10 min of exposure to NaCl. Similar differences in K^+^ efflux have been reported in barley (*H. vulgare*), wheat (*T. aestivum*) and alfalfa (*M. sativa*). This difference has been used as a selection criterion to distinguish salt-tolerant from salt-sensitive genotypes (Chen et al., 2005, 2007b,c; Smethurst et al., 2008), yet some plants, like rice, did not show such a correlation (Coskun et al., 2013). In our results, content and K^+^ efflux in roots (Figures 4, 5) were consistent with the observed differences in sensitivity between the varieties of habanero pepper (Figures 1, 2).

NaCl-induced K^+^ efflux may be through outward-rectifying K^+^ (KOR) channels activated by depolarization as demonstrated in barley (*H. vulgare*) and mangrove species (Chen et al., 2007b; Sun et al., 2009; Lu et al., 2013). The use of ion channels inhibitors in habanero pepper indicate that K^+^ efflux from the roots are likely mediated by KOR channels rather than by NSCC channels (Bojorquez-Quintal et al., in review). Furthermore, ROS and K^+^ deficiency have been associated with programmed cell-death (PCD) and necrosis (Anschütz et al., 2014; Demidchik, 2014; Shabala and Pottosin, 2014). Necrosis was observed in the roots of the Chichen-Itza variety (Figure 1), which have low ability to retain K^+^ (Figures 4, 5).

### Differences in H^+^ efflux in the roots of habanero pepper under NaCl stress

H^+^-ATPases generate an electrochemical gradient that maintains membrane potential and transports ions between the cytosol and the external medium. Under salt stress, NaCl universally induces H^+^ efflux in the roots of cereals such as *H. vulgare* and *T. aestivum*, halophytes such as *C. quinoa* and even in the model plant *A. thaliana* (Chen et al., 2007b; Cuin et al., 2008; Hariadi et al., 2011; Bose et al., 2013, 2014). In *C. chinense*, NaCl also induced H^+^ efflux in the mature root zone. H^+^ flux differed significantly between the varieties (Figure 6). NaCl rapidly induced the H^+^ efflux in both varieties of habanero peppers. It was suppressed by vanadate, the inhibitor of P-type H^+^-ATPase (Figure 7). H^+^ pumping activity of the plasma membrane H^+^-ATPase is essential for salt tolerance (Palmgren and Nissen, 2010). It has been suggested that the ability to K^+^ retain is related to the increase in H^+^-ATPase activity, primarily through a membrane potential repolarization (Bose et al., 2013, 2014).

Maintaining a more negative membrane potential during salt stress prevents the loss of K^+^ in the cytosol. In the salt-tolerant Arabidopsis relative species, *T. halophila*, a more negative membrane potential, correlated with a better K^+^ retention, was observed during salt stress (Volkov and Amtmann, 2006). In transgenic *A. thaliana* (HO, heme oxygenase), K^+^ retention was regulated by the increased H^+^-ATPase activity (Bose et al., 2013). A higher H^+^-ATPase activity maintained a more negative membrane potential and improved K^+^ retention in tolerant genotypes of *H. vulgare* (Chen et al., 2007b). In contrast, higher H^+^ transport activity was observed in the sensitive variety Chichen-Itza of *C. chinense* (Figure 6). Kinetics and magnitude of the H^+^ efflux coincides with that of the K^+^ efflux (Figure 5). Thus, it may be hypothesized that the descending phase of the H^+^ efflux reflects the condition of membrane potential repolarization, so that the leakage of K^+^ through KOR channels would be also reduced.

Furthermore, the plasma membrane H^+^-ATPase activity provides the driving force the Na^+^ extrusion via the Na^+^/H^+^ (SOS1) exchanger. In this work, we observed a massive accumulation of Na^+^ in the apoplast of the Chichen-Itza variety roots (Figure 9). These data suggest that SOS1 antiporter may be very active in the Chichen-Itza variety. However, Na^+^ efflux has a high energetic cost for the cell, especially keeping in mind a futile Na^+^ cycling between cytosol and apoplast. It may recruit the ATP available for other metabolic processes, thus, being detrimental to growth and yield. For this reason, the activation of H^+^-ATPase cannot be considered a permanent solution and might only be a temporal mechanism, as described by other studies (Ramani et al., 2006; Bose et al., 2013).

### Possible tolerance mechanism in peppers

**Figure 10:**
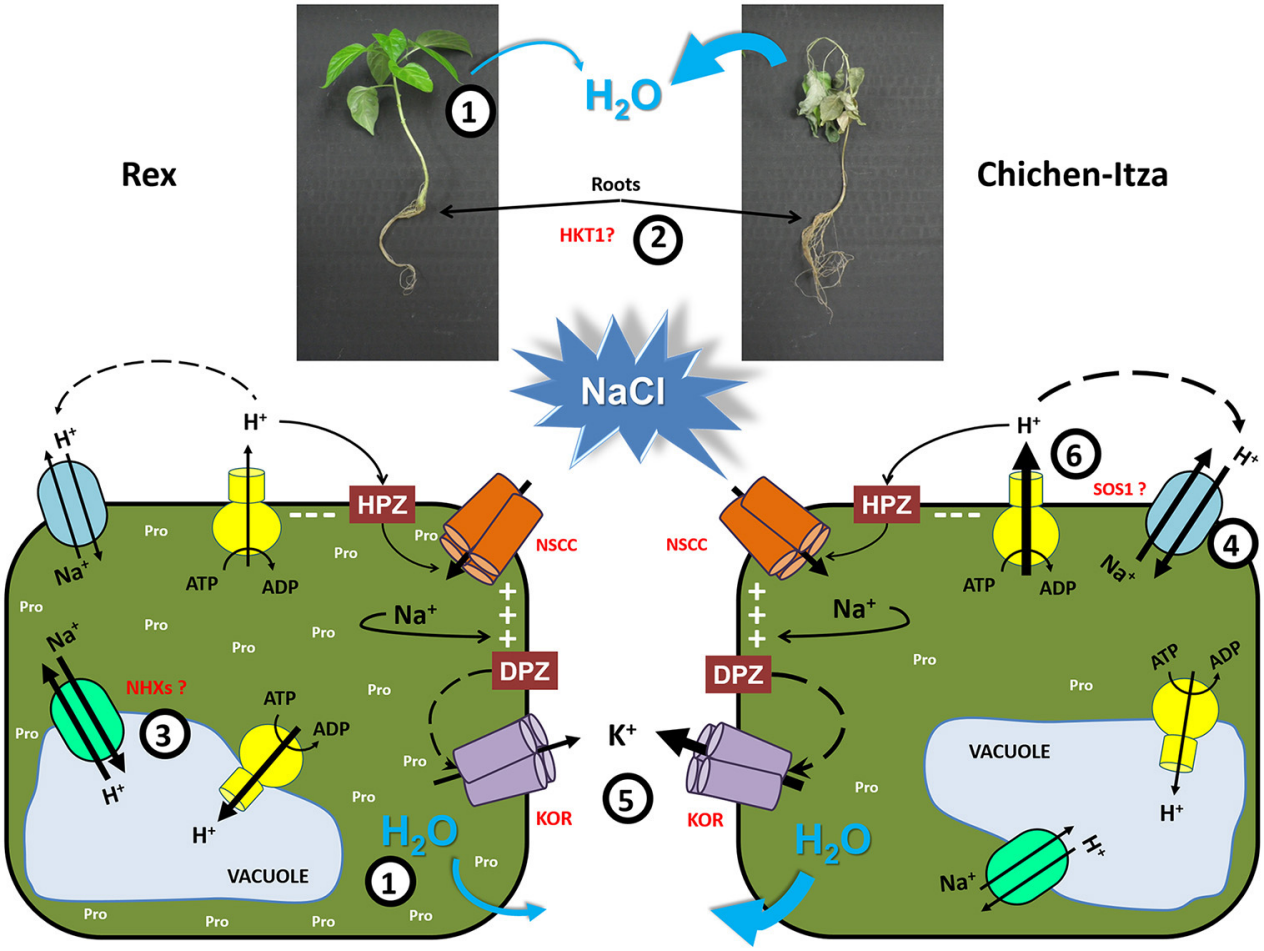
**Schematic model of habanero pepper plant tolerance mechanisms**. (1) Osmotic adjustment through the accumulation of compatible solutes as proline in leaves and roots to maintain the absorption and prevent the loss of water. (2) Efficient control of Na^+^ transport by confining this ion to roots, possibly through the recovery of Na^+^ from the xylem by HKT1 transporters to avoid transport to photosynthetic tissues. (3) Na^+^ compartmentalization in vacuole-like structures and small subcellular compartments, where it can act as an osmolyte, suggesting the involvement of vacuolar and endosomal NHX transporters. (4) Na^+^ extrusion or efflux from the cytosol to the apoplast by SOS1 exchangers. (5) Regulation of K^+^ homeostasis through K^+^ retention in roots is crucial in habanero peppers. (6) Increase in the H^+^-ATPase activity to generate the proton force for cell membrane repolarization or Na^+^ extrusion. (KOR), outward-rectifying K^+^ channels. HPZ is hyperpolarization and DPZ is depolarization.

Salt tolerance is a complex multigenic trait that involves many biochemical and physiological processes to achieve salt tolerance. In this paper, we demonstrate differences in salt sensitivity between two varieties of *C. chinense* Jacq, one of the five domesticated pepper species. We have analyzed several parameters of salt stress responses in both genotypes and their differences that may underlie their differential salt tolerance (Figure 10). One of salt tolerance mechanisms is the osmotic adjustment through the accumulation of compatible solutes (in this case, proline) in roots and leaves to maintain the absorption and prevent the loss of water (1). A second tolerance mechanism is the efficient control of Na^+^ transport by confining this ion to the roots, possibly through the recovery of Na^+^ from the xylem by HKT1 transporters (at low, moderate and high concentration of NaCl) to avoid transport to photosynthetic tissues (2). Furthermore, if the Na^+^ content in roots is high, this ion needs to be excluded from the cytosol to avoid toxicity. A third tolerance mechanism was observed in the tolerant variety (Rex), Na^+^ was efficiently compartmentalized into vacuole-like structures and small compartments which can act as osmolytes. This mechanism is possibly mediated by vacuolar and endosomal NHX antiporters (3). Additional mechanism appears to be involved in salt-sensitive variety (Chichen-Itza), which extrudes large amounts of Na^+^ into the apoplast (4). However, this mechanism appears to be less efficient due to its large energy cost. As in many other plant species, the regulation of K^+^ homeostasis through its retention in roots is crucial in habanero peppers (5), demonstrating the universality of this mechanism to the salt stress tolerance of crops.

### Conflict of interest statement

The authors declare that the research was conducted in the absence of any commercial or financial relationships that could be construed as a potential conflict of interest.

## Supplementary material

Figure S1 **K^+^ content of two varieties of *C. chinense* at different NaCl concentrations**. K^+^ content in the leaves **(A)** and roots **(B)** after treatment with salt. Forty-five-day-old seedlings cultivated in H1/5 for 7 days with 0, 50, 100 or 150 mM of NaCl. Bars represent averages for treatments with or without NaCl, ME ± SD (*n* = 3). The asterisk indicates statistically significant differences between varieties by treatment (*P* < 0.050, Tukey's test).

Figure S2 **Na^+^ content in Rex and Chichen-Itza varieties at different NaCl concentrations**. Na^+^ content in the leaves **(A)** and roots **(B)** after 7 days under salt stress conditions. Forty-five-day-old seedlings were cultivated in H1/5 with 0, 50, 100, and 150 mM of NaCl. Bars represent the average effect of the treatments with or without NaCl, ME ± SD (*n* = 3). The asterisk indicates statistically significant differences between varieties by treatment (*P* < 0.050, Tukey's test).

Figure S3 **Roots of control seedlings exhibit an absence of Sodium Green™ fluorescence**. Roots of untreated Rex **(A)** and Chichen-Itza **(B)** varieties stained with Sodium Green (SG), FM4-64, and DAPI. Images are representative of the analysis of three roots per treatment and variety.
