# Supplementary figures and images for: Mechanisms of salt tolerance in habanero pepper plants (Capsicum chinense Jacq.): Proline accumulation, ions dynamics and sodium root-shoot partition and compartmentation
Source: Front Plant Sci. 2014 Nov 12;5:605. doi: 10.3389/fpls.2014.00605 (PMC4228851; doi:10.3389/fpls.2014.00605)

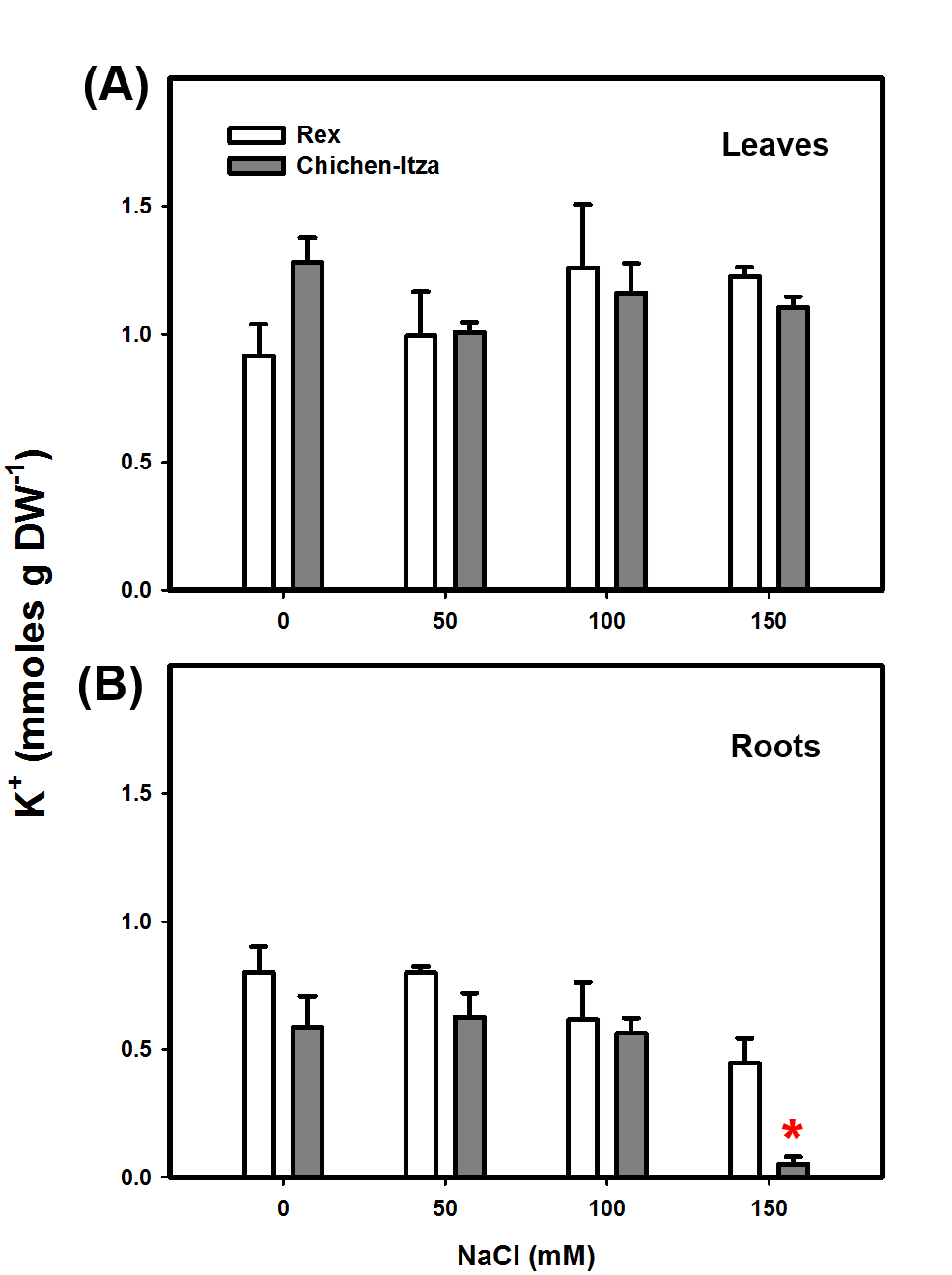

Supplement: Figure S1 — K+ content of two varieties of C. chinense at different NaCl concentrations. K+ content in the leaves (A) and roots (B) after treatment with salt. Forty-five-day-old seedlings cultivated in H1/5 for 7 days with 0, 50, 100 or 150 mM of NaCl. Bars represent averages for treatments with or without NaCl, ME ± SD (n = 3). The asterisk indicates statistically significant differences between varieties by treatment (P < 0.050, Tukey's test). [file Image1.TIF]

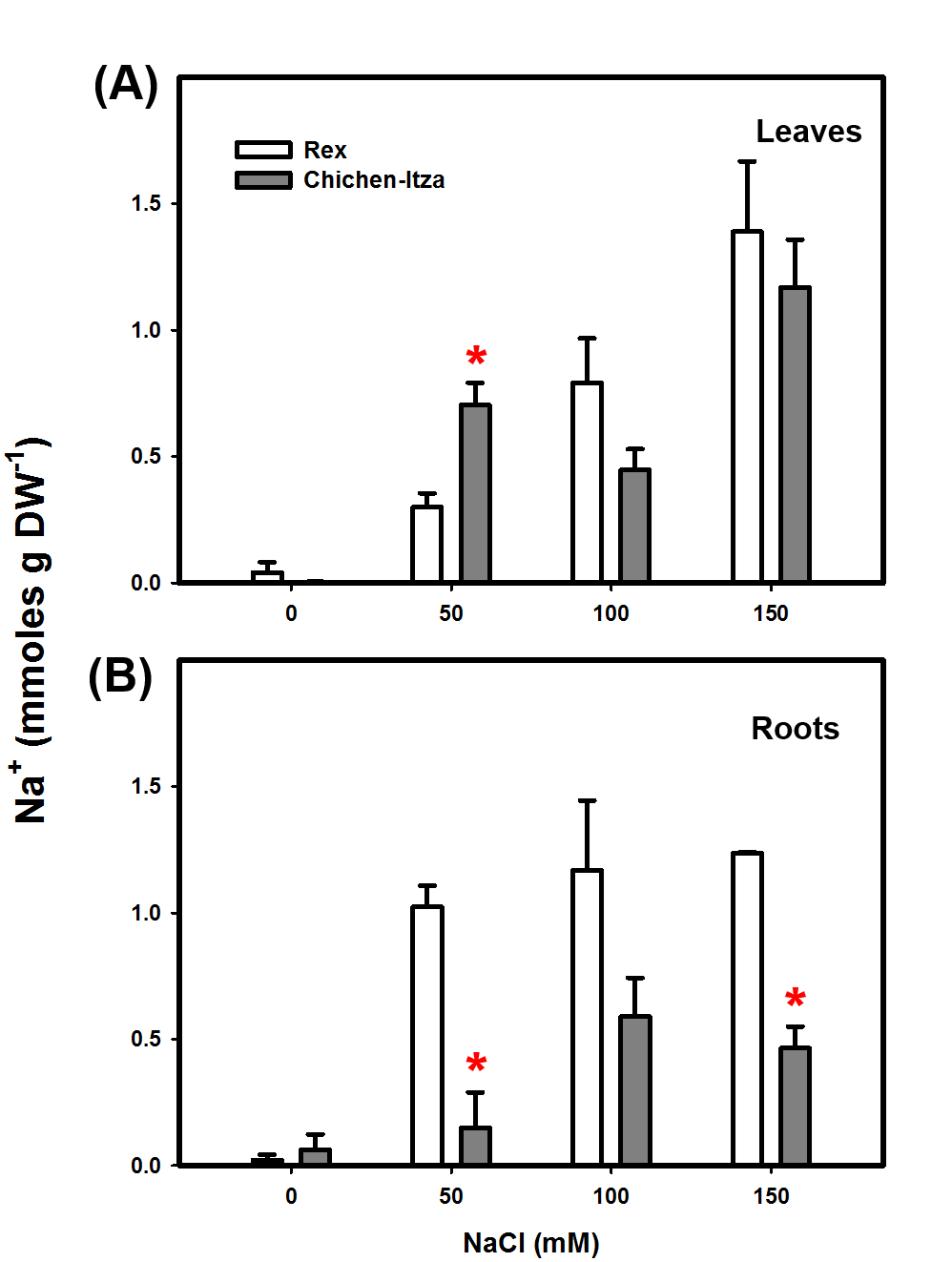

Supplement: Figure S2 — Na+ content in Rex and Chichen-Itza varieties at different NaCl concentrations. Na+ content in the leaves (A) and roots (B) after 7 days under salt stress conditions. Forty-five-day-old seedlings were cultivated in H1/5 with 0, 50, 100, and 150 mM of NaCl. Bars represent the average effect of the treatments with or without NaCl, ME ± SD (n = 3). The asterisk indicates statistically significant differences between varieties by treatment (P < 0.050, Tukey's test). [file Image2.TIF]

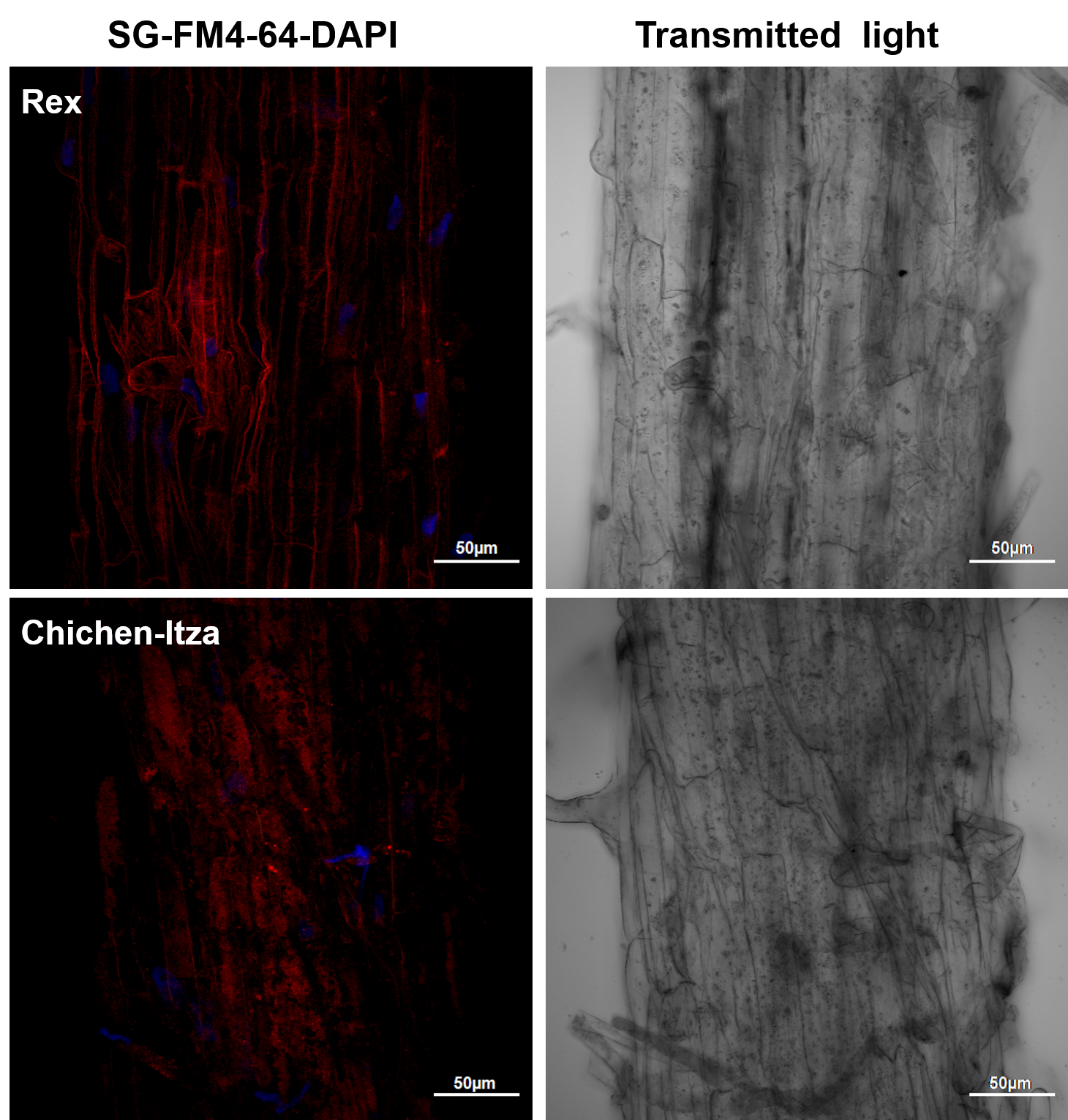

Supplement: Figure S3 — Roots of control seedlings exhibit an absence of Sodium Green™ fluorescence. Roots of untreated Rex (A) and Chichen-Itza (B) varieties stained with Sodium Green (SG), FM4-64, and DAPI. Images are representative of the analysis of three roots per treatment and variety. [file Image3.TIF]
